# Case Report: Congenital Intracranial Kaposiform Hemangioendothelioma Treated With Surgical Resection

**DOI:** 10.3389/fsurg.2022.831190

**Published:** 2022-04-01

**Authors:** Yingjie Cai, Jiayi Li, Wei Yang, Nan Zhang, Hailang Sun, Weiping Zhang, Ming Ge

**Affiliations:** ^1^National Center for Children's Health, Department of Neurosurgery, Beijing Children's Hospital, Capital Medical University, Beijing, China; ^2^Graduate School, National Center for Children's Health, Beijing Children's Hospital, Capital Medical University, Beijing, China; ^3^National Center for Children's Health, Department of Pathology, Beijing Children's Hospital, Capital Medical University, Beijing, China

**Keywords:** intracranial, Kaposiform hemangioendothelioma, congenital, rare, neurosurgery

## Abstract

**Background:**

Kaposiform hemangioendothelioma (KHE) is a locally aggressive but non-metastatic vascular neoplasm. Most studies have been restricted to small case series of limited generalizability. Intracranial KHE is extremely rare with only three cases reported in the literature. Here, we report a case of congenital intracranial KHE who underwent surgical resection, and no lesion recurrence was seen during the follow-up period of 13 months.

**Case Description:**

A 2-month-old boy initially presented with a left temporal mass following birth. Antenatal ultrasound at 36 weeks of gestation demonstrated a hyperechoic signal present in the left frontal lobe, with clear borders and irregular morphology. There were neither cutaneous abnormalities nor other neurologic examination abnormalities. No laboratory abnormality was identified. Computed tomography (CT) scans suggested that a massive hematoma was noted under the left frontal skull plate, with a little subdural hemorrhage in the adjacent temporal area. The adjacent meninges enhanced and thickened on contrasted T1 magnetic resonance (MR). After the multidisciplinary diagnostic assessment, the surgery was performed by the left frontotemporal craniotomy approach. The operation was extremely difficult. We completely removed the tumor, and the involved dura and brain tissue were resected with the lesion in a piecemeal fashion. On postoperative-day (POD) 3 and POD 14, the head CT re-examination revealed that cerebral perfusion improved gradually. The MRI of 6- and 12-month after operation showed no local recurrence or metastasis.

**Conclusions:**

Intracranial KHE is difficult to diagnose early and the prognosis has been uniformly poor. We supposed that meticulous intraoperative hemostasis is the key for a successful operation, and the radical resection of the tumor and involved structures are essential to reduce recurrence.

## Introduction

Kaposiform hemangioendothelioma (KHE) is an extremely rare, locally aggressive but non-metastatic vascular neoplasm that always arises during infancy or early childhood. The incidence of KHE is about 0.1 in 100,000 children (1). The clinical features of KHE are varied, about 70% of children with KHE may develop Kasabach–Merritt phenomenon (KMP), known as life-threatening thrombocytopenia and consumptive coagulopathy. In addition, ~88% of patients with KHE may develop skin manifestations, commonly occurring in the head, neck, and extremities, which presents as symptomatic, progressively enlarging masses that may warrant surgical excision (2–4). However, intralesional hemorrhage and hematoma formation may lead to severe anemia and hemodynamic instability, thus a surgery could potentially be traumatic without adequate caution.

To date, most KHE studies have been restricted to case reports and the small case series of limited generalizability and intracranial KHE are extremely rare with only three cases reported in the literature, and no cured cases were reported (5–7). Here, we report a case of congenital intracranial KHE who underwent surgical resection, no lesion recurrence was seen during the follow-up period of 13 months. Relevant literature has been reviewed, and the clinicopathological features, diagnosis, treatment, and prognosis have been discussed. To the best of our knowledge, this is the first report describing a case of congenital intracranial KHE as well as the first case with no recurrence for more than 1 year.

## Case Presentation

### History and Examination

**Figure 1:**
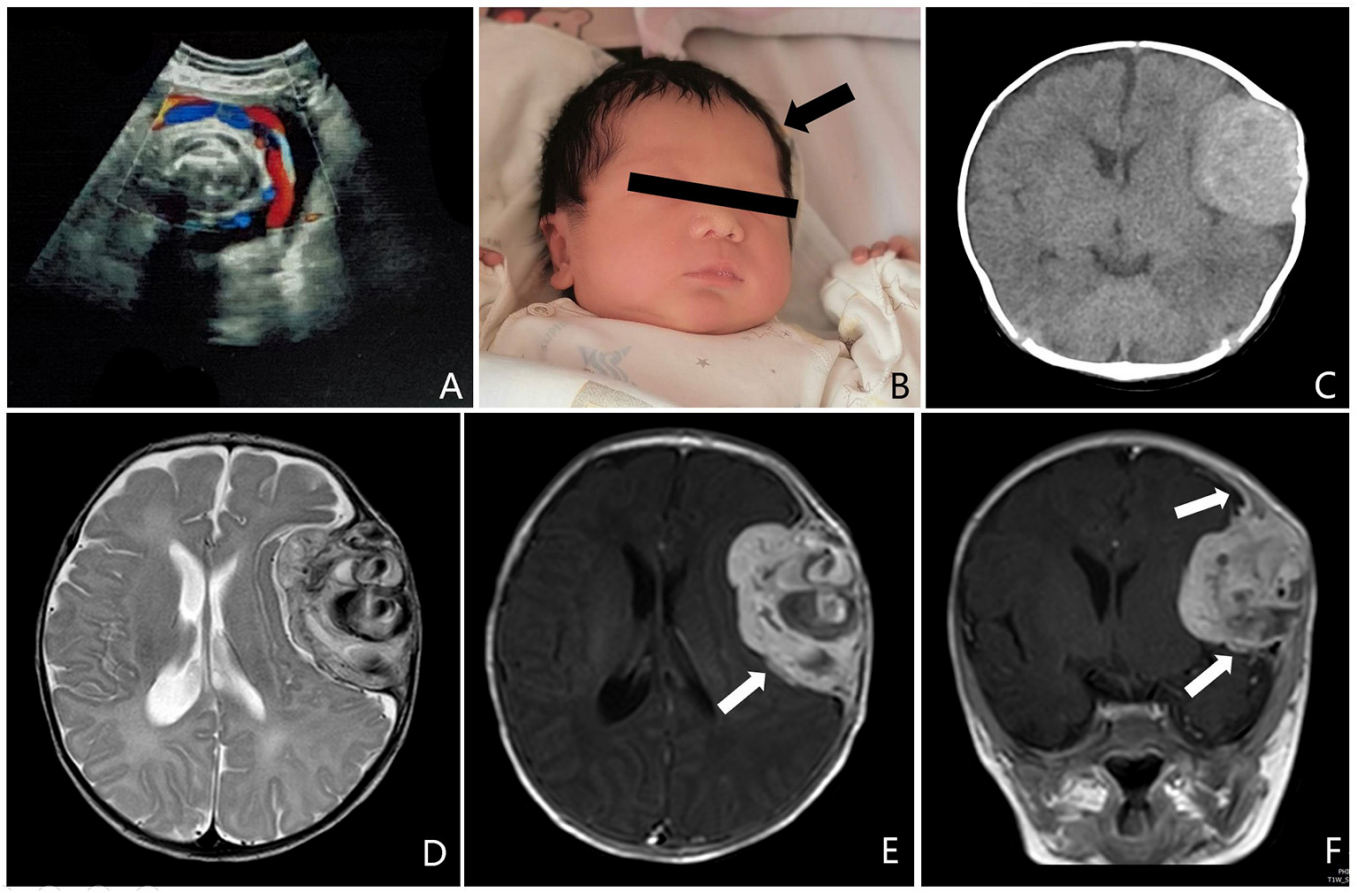
**(A)** Antenatal ultrasound at 36 weeks of gestation demonstrated a hyperechoic signal was present in the left frontal lobe, with clear borders, and irregular morphology. Low resistance blood flow signal was seen within the abnormal entity. **(B)** At the age of 15 days, a slight bulge was observed on the left forehead (arrow). **(C)** Preoperative computed tomography (CT) scans suggested that a massive hematoma was noted under the left frontal skull plate, with a little subdural hemorrhage in the adjacent temporal area. The circumambient brain parenchyma is obviously compressed and the affected skull bulges outward. **(D–F)** Preoperative brain magnetic resonance (MR) examination found a huge epidural mass under the left frontal skull, with a heterogeneous internal signal, and multiple patchy low signals. The adjacent meninges enhanced and thickened on T1 MRI after enhancement (arrow).

A 2-month-old boy initially presented with a left temporal mass following birth. Antenatal ultrasound at 36 weeks of gestation demonstrated that the biparietal diameter was 9.64 cm, the head circumference was 34.71 cm. The ventricles are in normal shape, with no signs of cerebral hernia. A hyperechoic signal was present in the left frontal lobe, with clear borders and irregular morphology. Low resistance blood flow signal was seen within the abnormal entity (Figure 1A). Surveillance was recommended because it was difficult to have a definite pathological diagnosis before childbirth. The boy was delivered at 38 weeks gestation, and a slight bulge was observed at the left forehead and gradually increased with age (Figure 1B). There was no family history of hereditary disease, malignancy, and other known neurologic conditions. On physical examination, the head circumference was 41 cm; the anterior fontanel was soft, there is an ~3.5 cm × 4.5 cm × 4.5 cm bony bump on the top of the left frontal area. There were no cutaneous abnormalities, such as ecchymosis, swelling, erythema, and other evidence of neurocutaneous lesions. There was no hepatosplenomegaly. Other neurologic examinations showed no abnormalities. No laboratory abnormality was identified, such as a coagulation dysfunction or evidence of systemic disease. Computed tomography (CT) scans suggested that a massive hematoma was noted under the left frontal skull plate, with a little subdural hemorrhage in the adjacent temporal area. The circumambient brain parenchyma is obviously compressed and the affected skull bulges outward. Hyperplasia, compression, and erosion are seen in the upper and lower part of the skull, respectively (Figure 1C). The brain magnetic resonance (MR) examination found a huge epidural mass under the left frontal skull, with a heterogeneous internal signal, and multiple patchy low signals. The adjacent meninges enhanced and thickened on post-contrast T1 MRI (Figures 1D–F).

### Surgery

**Figure 2:**
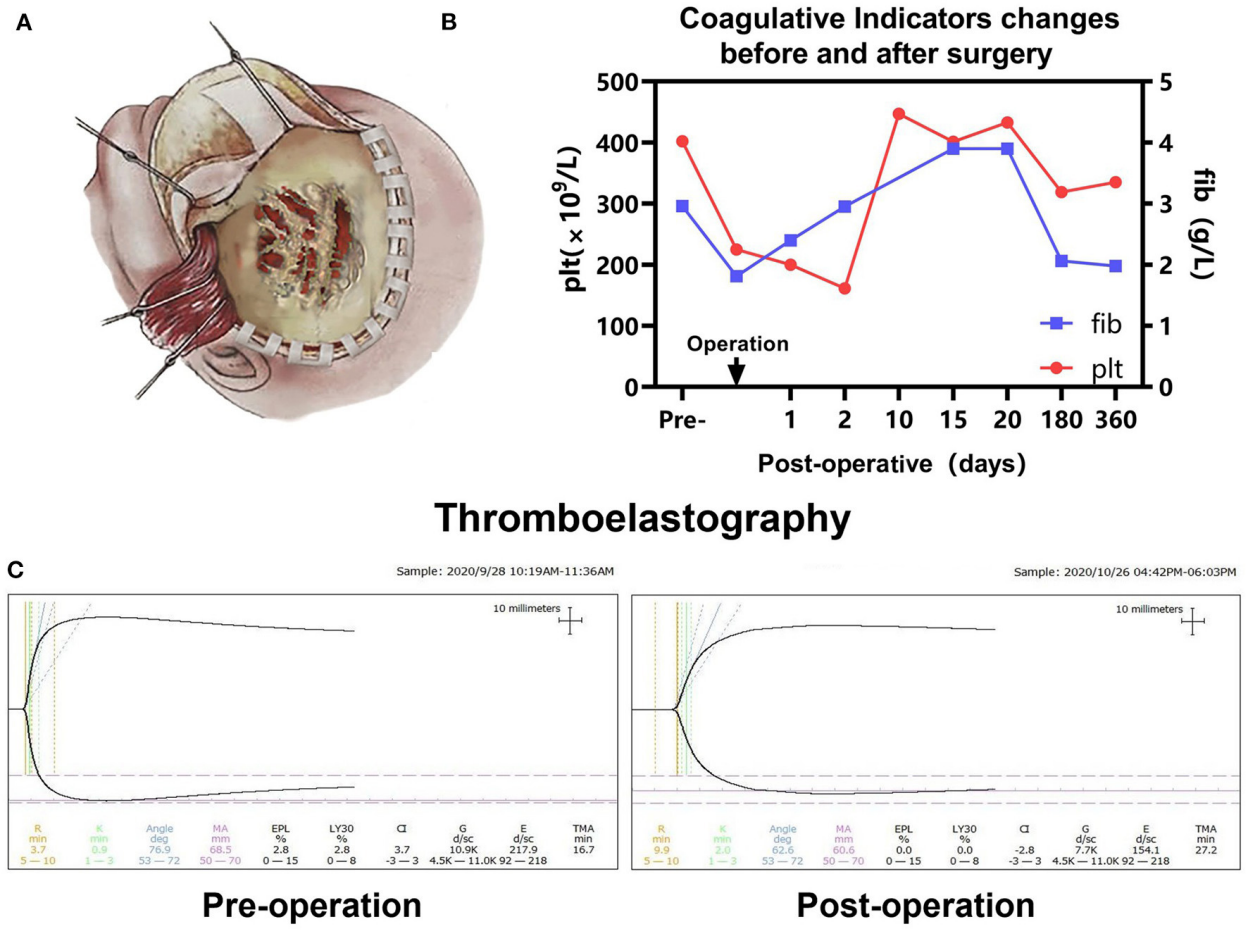
**(A)** Schematic diagram of the left frontotemporal craniotomy approach in our case, the seeping blood, worm-like changes were observed on the surface of the skull, the skull was invaded seriously by tumor and getting thinner. **(B)** Pre- and postoperative platelet (Plt) and fibrinogen (Fib) were monitored. **(C)** Pre- and postoperative thromboelastography revealing that no coagulopathy was observed.

In consideration of the contextual information of the patient and a multidisciplinary diagnostic assessment, the possibility of malignant tumors was considered. The blood was well-prepared to prevent profound intraoperative hemodynamic instability. A surgery was performed by the left frontotemporal craniotomy approach. During the operation, the seeping blood, worm-like changes were observed on the surface of the skull (Figure 2A). When removing the skull, it can be seen that the skull was invaded seriously by the tumor and was getting significantly thinner. The bone flap was removed due to close adhesion to the tumor. The tumor presented with dark red color and was abundant with blood supply. It was firmly attached to the surface of the dura, partly destroyed the dura, and proceeded forward to the brain tissue. We completely removed the tumor, the involved dura and brain tissue were resected with the lesion in a piecemeal fashion. During the procedure, massive vascular abnormalities in the left lateral fissure area were observed, poorly demarcated with mass lesions. The estimated blood loss was 1,200 ml, 8 units of whole blood, 500 ml frozen plasma transfusion, and 1 unit of platelet were transfused intra-operatively. After the surgery, the patient was in a coma with pupillary asymmetry (left pupil 4 mm and right 2 mm). Then, the patient was transferred to the intensive care unit (ICU) for postoperative intensive care.

### Histopathological Findings

**Figure 3:**
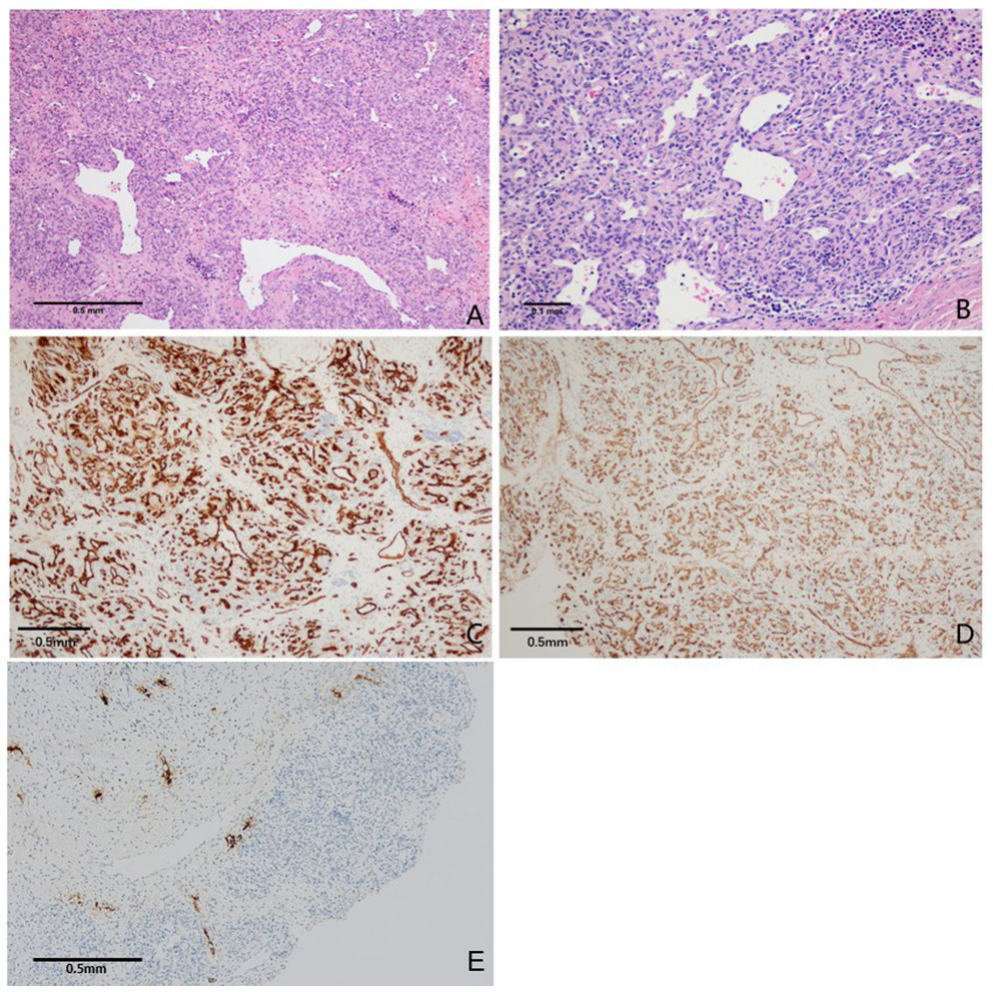
Photomicrographs of tumor tissue. **(A)** Tumor cells were diffusely distributed, partly showed lobulated shape with mild collagenization of the stroma, a few staghorn-like vascular channels were observed (H&E, original magnification × 100). **(B)** Tumor cells are smaller in size, lack atypia. The neoplastic cells were focally arranged in a fascicular pattern in the context of infiltration of lymphocytes and histiocytes (H&E, original magnification × 200). **(C,D)** The tumor cells were strongly positive for CD31 and CD34. Vascular endothelial cell proliferation was observed (immunoperoxidase × 200). **(E)** Immunohistochemical staining for D2-40 expression was positive (immunoperoxidase × 100).

Pathological histology findings revealed that the tumor cells were arranged as flaky, exhibited a nodular scattered distribution. The tumor showed infiltrative growth with invasion to the surrounding cranium bone. Tumor cells showed oval- to short-spindle-shaped, possessed vesicular nuclei without division, and the weak staining of the tumor cell cytoplasm was observed. The interstitium between the tumor cells is fine with small capillaries and the vessel lumen was dilated. The neoplastic cells were focally arranged in a fascicular pattern in the context of infiltration of lymphocytes and histiocytes. This is accompanied by flake bleeding lesions, multifocal calcifications, and hemosiderin deposition (Figures 3A,B).

Immunohistochemically, the neoplastic cells are diffusely positive for CD31 and CD34, partially positive for D2-40 and SMA. The Ki67 proliferative index was ~20%. These histopathological findings were consistent with the diagnostic criteria of KHE (Figures 3C–E).

### Postoperative Course

**Figure 4:**
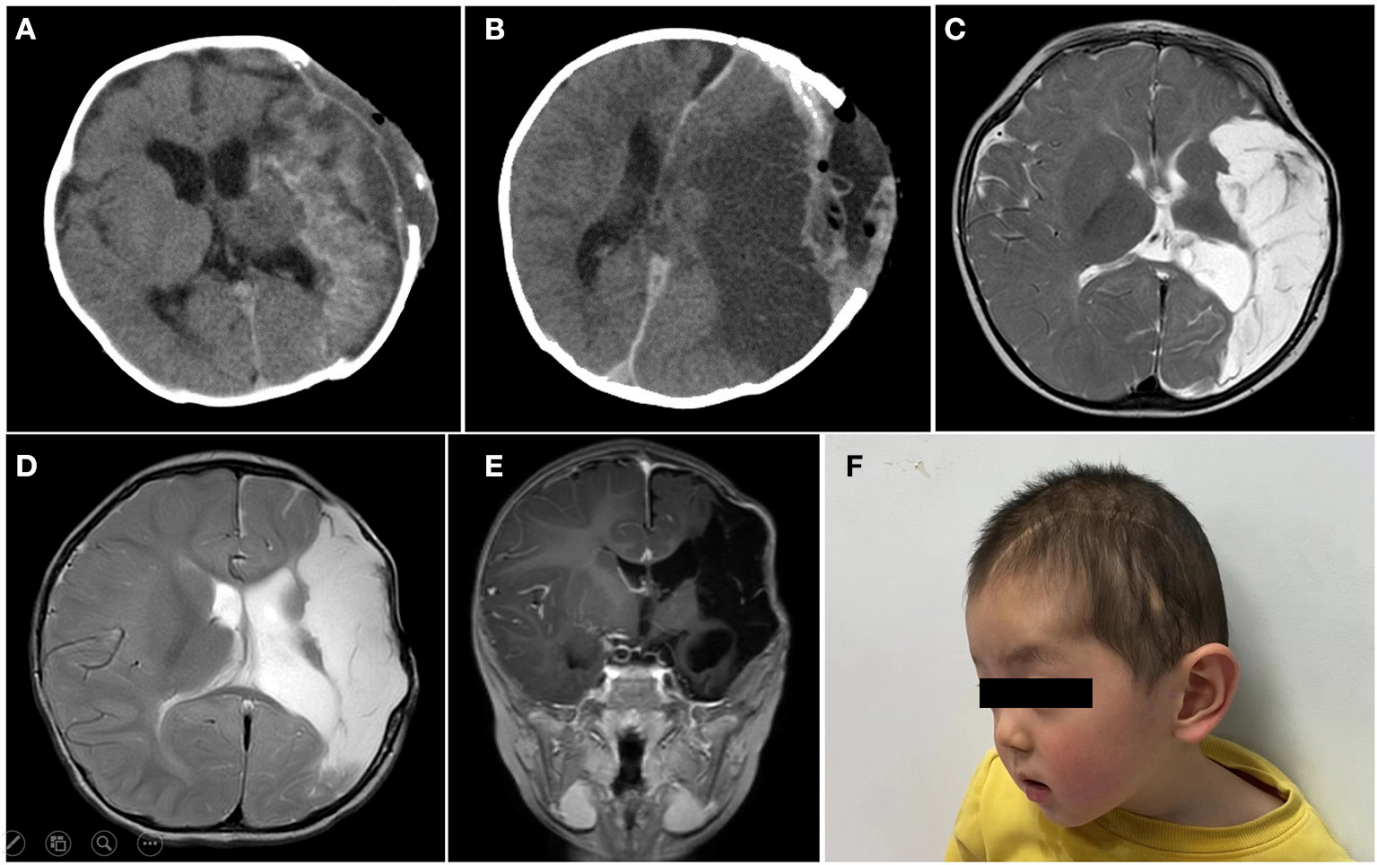
On postoperative-day (POD) 3 **(A)** and POD 14 **(B)**, head CT re-examination revealed no tumor residues. The brain MR examination of 6 months **(C)**, 12 months **(D,E)** postoperatively, suggested ischemic and liquefaction changes in the part of left frontal and temporal lobes and basal ganglia. No local recurrence or metastasis was observed. **(F)** Briefly, 12 months postoperatively, a skull defect can be seen in the forehead of the patient, and the spontaneous facial expressions are symmetrical but involuntary salivation was observed.

The patient was in intensive care for 4 days. As a result of the postoperative examination and the laboratory findings, the patient was diagnosed with temporal lobe uncinate herniation, hemorrhagic shock, coagulation disorders, and acute electrolyte imbalance. Pre- and post-operative thromboelastography revealing no coagulopathy was observed (Figure 2C). Ramsay Sedation Score was 6, Glasgow Score was 3T (E1VTM1), FOUR score was 2 (E0M0B2R0). The patient lost spontaneous respiration and the invasive mechanical ventilator was needed. Due to the blood-loss anemia and thrombocytopenia, 2 units of whole blood and 100 IU prothrombin complex concentrate were transfused postoperatively. After multiple salvage treatments, the vital signs of the patient were within normal limits. Pupillary reflex returned to normal and an autonomous cardiac rhythm and respiration were restored. On postoperative-day (POD) 3 and POD 14, head CT re-examination revealed no tumor residues (Figures 4A,B). The patient was discharged 15 days following surgery.

### Follow-Up

The patient was followed up over a duration of 13 months at the outpatient Department. Platelet and coagulation functions were monitored regularly (Figure 2B). The brain MRI (6 months after operation) suggested ischemic and liquefaction changes in the part of left frontal and temporal lobes and basal ganglia. Wallerian degeneration was observed in the thalamus and cerebral peduncles. There are no signs of any tumor recurrence (Figure 4C). The MRI of 12 months after operation showed no significant change compared with the previous, no local recurrence or metastasis was observed (Figures 4D,E). The follow-up examination at 1 year after surgery, the mental status of patient was subsequently improved, but still had right-sided hemiparesis with the muscle strength of 2/5 in the right upper limb and muscle strength of 3/5 in the right lower limb. A large skull defect can be seen in the forehead, and the spontaneous facial expressions were symmetrical but involuntary salivation was observed (Figure 4F). The cranioplasty was scheduled. Moreover, follow-up with pediatric neurology patient examination showed motor developmental and speech delays, with the Gross Motor Function Measure (GMFM) Score of 53/264. At the age of 15 months, the patient was able to ambulate with help (Supplementary Material), no language deficits were observed.

## Discussion

Kaposiform hemangioendothelioma was first reported in 1993 and the clinical, imaging, and pathological features were systematically characterized by Ji et al. in 2018 (1, 5). As reported in literature, the estimated prevalence of KHE is 0.7–0.91 in 100,000 children and over 50% of KHE lesions were present at birth (1, 6). It was categorized by the International Society for the Study of Vascular Anomalies (ISSVA) as locally aggressive or borderline vascular tumors (7). The neoplastic spindle cells express the vascular markers CD31 and CD34, the vascular endothelial growth factor receptor-3 (VEGFR-3), and the lymphatic markers D2-40 and PROX1, suggesting that KHE may derive from the lymphatic endothelium (5, 8, 9). The lesions of KHE may involve superficial and deep soft tissues and, rarely, the retroperitoneum, mediastinum, and internal organs, most of them may grow rapidly and a small subgroup remained unchanged (6), however, the intracranial lesion of the tumor was rarely seen. Based on morphological features, Ji et al. classified KHE lesions as superficial, mixed (cutaneous lesions with deep infiltration), and deep (lesions without cutaneous involvement), 12% of patients lacked cutaneous involvement (10). The KMP is a life-threatening complication, characterized by profound thrombocytopenia and consumption coagulopathy, the mechanism is so far unidentified. One hypothesis is that the abnormal endothelium and convoluted architecture of the tumor vasculature promote platelet adhesion and trapping (9). Platelet aggregation and activation result in thrombocytopenia, the consumption of fibrinogen, and ongoing fibrinolysis, leading to intralesional bleeding and tumor enlargement (11). KMP occurs in ~70% of the cases of KHE, nevertheless, the presence of KMP was not reported in intracranial KHE cases.

The diagnosis of KHE is based upon the combination of clinical, histologic, and imaging features (12). As mentioned, however, the presence of a highly vascular tumor together with thrombocytopenia and coagulopathy greatly increases the risk of a biopsy. It is difficult to diagnose definitively in the case of individuals without characteristic clinical presentation and laboratory findings. The imaging differential diagnosis includes intracranial neurofibromatosis and intracranial metastatic tumor from neuroblastoma. Both of them are located under the skull plate, presenting an isodensity or slightly high-density round lesion on CT scan, and a long T1 signal and long T2 signal on MRI, with obvious enhancement after contrast. Differential diagnosis of these lesions is required by histopathological examination. The morphologic differential diagnosis of KHE, such as tufted angioma, infantile hemangiomas, congenital hemangiomas, kaposiform lymphangiomatosis, infantile myofibromatosis, infantile fibrosarcoma, angiosarcomas, venous malformations, and kaposi sarcoma (13–16). The histopathologic appearance of KHE is characterized by lobules or sheets of tightly packed spindled or more rounded endothelial cells, with an infiltrative pattern in the dermis, subcutaneous fat, and muscles. As for immunohistochemical analysis, endothelial cells express the vascular markers CD31, CD34, and the lymphangiogenesis marker, vascular endothelial growth factor receptor-3. For differential diagnosis, infantile hemangiomas exhibit the lobulated masses of proliferating endothelial cells and a strong expression of GLUT-1. Rapidly involuting congenital hemangiomas differ in having the lobules of capillary proliferations embedded in a fibrous stroma without spindle cell or lymphatic ectasias. Kaposiform lymphangiomatosis is characterized by diffuse lymphatic malformation with the focal areas of “kaposiform” spindle cells vs. the diffuse nature of the spindle cells in KHE. The histology of infantile myofibromatosis reveals well-circumscribed nodules of round-shaped and spindle-shaped cells with variable cytologic atypia and haphazard perivascular orientation and an increased number of thin-walled, branching “staghorn” vessels. Kaposi sarcoma presents extensive vascular proliferation in the dermis with multiple dilated vascular spaces and solid cords and fascicles of spindle cells arranged between the jagged vascular channels. In the present case, the results of H&E staining and immunohistochemical analysis confirmed the diagnoses of KHE.

**Figure 5:**
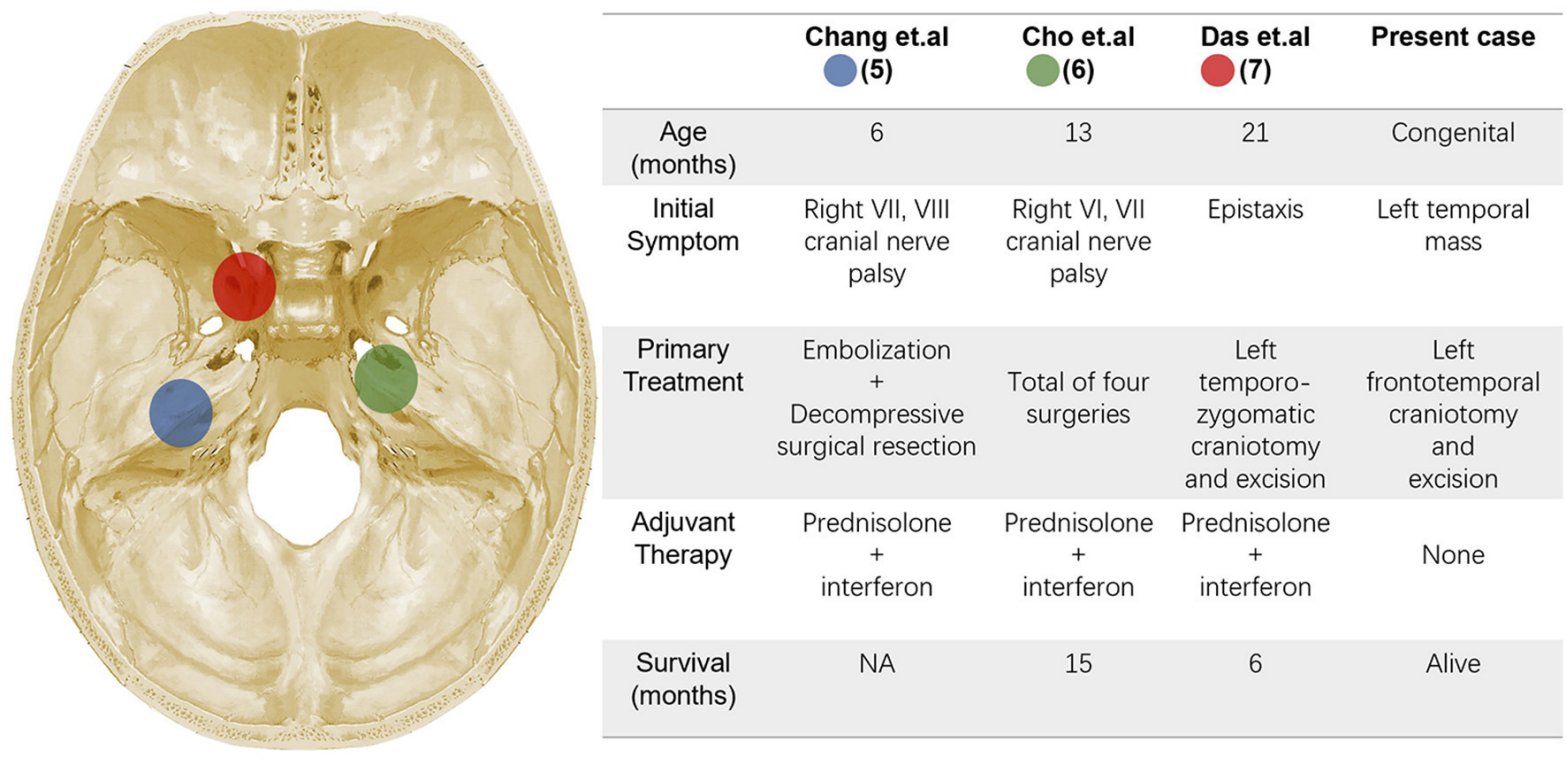
The different colors indicate KHE lesions locations of the literature.

To the best of our knowledge, there have only been 3 cases of patients with intracranial KHE previously reported in the literature, and no congenital case was reported (17–19) (Figure 5). All the 3 cases reported in the literature are boys, the age at onset was 6–21 months, no KMP was observed and no evidence of gross coagulation abnormalities was noted. The tumors near the midline and clinical presentation lack specificity, therefore failing to clarify the diagnosis before surgery. As for our patient, the tumor was previously detected in the prenatal ultrasonography, and similar to what has been reported in the literature, progressive enlargement of the tumor is the only clinical expression, no laboratory abnormality was identified. Preoperative imaging examination revealed a rich blood supply of the tumor parenchyma, thus it was too dangerous to perform a biopsy of tumor tissue or bone. With respect to the treatment in literature, surgical resection combined with postoperative hormone plus interferon (IFN) therapy is the mainstay method. However, the prognosis was not ideal, two died of postoperative pneumonia and sepsis, and no data on the clinical outcomes and follow-up of the third patient were described. Cho et al. reported a KHE case, the lesion originated from the tentorium cerebelli, extending from the right cerebellopontine angle to the middle cranial fossa. Most of the mass was removed except for the medial capsule in the primary operation and the patient underwent adjuvant IFN-α and prednisolone therapy. However, the patient was found to have a recurrence for 3- and 6-month following the first surgery, which caused the blindness of the right eye. After four operations, the patient died due to pneumonia and sepsis. Based on its clinical course and pathological characteristics, the author argued to propose “variant KHE” as a malignant form (18). For the present case, the tumor was predominantly located between the skull and dura, it is no doubt that complete resection of the tumor with resection of the involved skull and dura is the key to prevent relapse.

When the intracranial tumor can affect the vascular functions and cause corresponding signs or symptoms, treatment protocols should prioritize the option for radical surgery, while the rich blood supply of the KHE lesion greatly increased the risk of intraoperative profuse bleeding. Prior studies demonstrated that embolization may help decrease the blood flow to the lesion, preventing excessive bleeding (20–22), but Chang et al. argued that for intracranial KHE lesion, preoperative embolization cannot effectively reduce intraoperative bleeding despite near-total devascularization was observed on postembolization angiography (17). In the present case, intraoperative hemostasis is a serious challenge. Massive intraoperative bleeding, adhesions, altered anatomy from previous surgery, and the urgency of the situation prevent us from identifying anatomic angioarchitectures during the surgery. Mechanical compression was attempted first followed by the placement of hemostatic gauze and a drainage tube was placed under the dura mater. Besides, we believe that under the premise of protecting the main intracranial blood vessels, the lesion should be completely removed as much as possible, which is also an important factor for successful hemostasis. The profound intraoperative hemodynamic instability led to a life-threatening condition, and the clinical condition of patient gradually improved mainly attributed to multidisciplinary care. Although a 1-year period of follow-up shows no recrudescence or metastasis, we speculate that blood loss and vessel injury were the main reason for postoperative cerebral infarction.

For tumors that are not resectable, are symptomatic, and have a high risk of functional compromise, there is no consensus on the pharmacologic approach. For patients that are not complicated by KMP, oral prednisone or prednisolone is currently considered to be the first-line treatment, and aspirin can be given as adjunctive therapy (23). Other treatments, such as vincristine, sirolimus, propranolol, interferon-alpha, and ticlopidine, have been used alone or in combination (12, 24, 25). The latest randomized controlled trial revealed that the use of short-term prednisolone treatment plus sirolimus therapy can normalize hematologic parameters, reduce lesion mass promptly, and prevent long-term sequelae, suggesting that sirolimus plus prednisolone should be considered as a valid treatment for KHE with KMP (26). For intracranial cases, as reviewed, prednisolone and interferon-alpha were used as the postoperative adjuvant therapy, which has not resulted in a superior prognosis, where two cases succumbed to the sepsis instead of tumor recurrence. Thus, the close monitoring of serum levels should be emphasized, and dose adjustments should be made accordingly, based upon the tumor response to treatment and acceptable therapy-related toxicity. In the present case, taking into account the age of the child and the opinions of the parents, we chose to temporarily follow-up without hormone therapy postoperatively. Although the results of long-term follow-up remain unknown, a 1-year period of follow-up shows no recrudescence or metastasis.

## Conclusion

Intracranial KHE is extremely rare, difficult to diagnose early, and prognosis has been uniformly poor. We report a case of congenital intracranial KHE who underwent surgical resection, no recurrence was observed during the follow-up period of 13 months. We supposed that meticulous intraoperative hemostasis is the key for a successful operation, and radical resection of the tumor and involved structures are essential to reduce recurrence.

## Data Availability Statement

The raw data supporting the conclusions of this article will be made available by the authors, without undue reservation.

## Ethics Statement

The studies involving human participants were reviewed and approved by the Ethics Committee of Beijing Children's Hospital, Capital Medical University, National Center for Children's Health (IEC-C-006-A04-V.06). Written informed consent from the participants' legal guardian/next of kin was not required to participate in this study in accordance with the national legislation and the institutional requirements.

## Author Contributions

YC and JL wrote the first draft of the article and were responsible for the overall content. NZ evaluated the pathological histology findings. WY evaluated the radiological images and contributed mainly to the discussion of the cases. HS performed the surgical treatment on the patient and contributed significantly to the patient's discussion. WZ and MG supervised the study. All authors contributed to the article and approved the submitted version.

## Conflict of Interest

The authors declare that the research was conducted in the absence of any commercial or financial relationships that could be construed as a potential conflict of interest.

## Publisher's Note

All claims expressed in this article are solely those of the authors and do not necessarily represent those of their affiliated organizations, or those of the publisher, the editors and the reviewers. Any product that may be evaluated in this article, or claim that may be made by its manufacturer, is not guaranteed or endorsed by the publisher.
